# Serum Neurofilament Light Chain Levels May Be a Marker of Lower Motor Neuron Damage in Amyotrophic Lateral Sclerosis

**DOI:** 10.3389/fneur.2022.833507

**Published:** 2022-02-23

**Authors:** Linjing Zhang, Tuo Ji, Chujun Wu, Shuo Zhang, Lu Tang, Nan Zhang, Xiangyi Liu, Dongsheng Fan

**Affiliations:** ^1^Department of Neurology, Peking University Third Hospital, Beijing, China; ^2^Beijing Municipal Key Laboratory of Biomarker and Translational Research in Neurodegenerative Diseases, Beijing, China; ^3^Department of Neurology, Zhengzhou University First Affiliated Hospital, Zhengzhou, China; ^4^Department of Neurology, Beijing Tiantan Hospital, Capital Medical University, Beijing, China; ^5^Key Laboratory for Neuroscience, National Health Commission/Ministry of Education, Peking University, Beijing, China

**Keywords:** NfL, amyotrophic lateral sclerosis, axonal degeneration, CMAPs, EMG - electromyogram

## Abstract

Objectives The aims of this study were to investigate whether serum neurofilament light chain (NfL) levels were correlated with the severity of the axonal degeneration of lower motor neurons (LMNs) in the early symptomatic phase of amyotrophic lateral sclerosis (ALS). Methods In this prospective study, the serum samples used for NfL measurement were obtained from 103 sporadic ALS outpatients within 2 years of disease duration. The severity of axonal degeneration was assessed by assessing the decrease in the compound muscle action potentials (CMAPs) within a 1-month interval from serum sampling. Results The NfL levels showed a significant positive correlation with the relative score as a proxy for the axonal damage of LMNs in patients with ALS (coefficient: 0.264, *p* = 0.009). Furthermore, this correlation became stronger (coefficient: 0.582, *p* = 0.037) when estimated only among patients with disease subtypes that involve only LMNs, that is, patients with flail arm or leg syndrome (FAS or FLS). The levels of NfL increased with the severity of axonal damage of LMNs (F = 6.694, *P* = 0.0001). Conclusions Serum NfL levels mirrored the severity of the axonal degeneration of LMNs, particularly in patients with signs of predominant LMN involvement. These results may have a profound effect on the selection of patients and the monitoring of treatment efficacy in future disease-modifying clinical trials.

## Introduction

Amyotrophic lateral sclerosis (ALS), which selectively affects upper and lower motor neurons (MNs), is a fatal neurodegenerative disorder for which an efficacious therapy is urgently needed (1). In recent years, neurofilament light chain (NfL) has emerged as the most promising biomarker for use in diagnosis, prognosis, and monitoring disease progression and response to pharmacological intervention; thus, NfL may allow a deeper understanding of pathophysiological mechanisms and potential effective therapies for ALS (2–5). Specifically, a higher level of NfL is usually associated with faster disease progression and predicts a poorer prognosis (4). However, Gaiani et al. (4) correlated a higher cerebrospinal fluid (CSF) NfL level with subtypes of MN disorders, revealing that patients with upper motor neuron-dominant (UMND) ALS presented with high CSF levels of NfL, despite the well-known superior prognosis associated with this subtype (6, 7). This puzzling finding that UMND ALS patients have longer survival despite their higher CSF NfL levels indicates that in patients with UMND ALS, NfL levels are probably not a reliable and effective prognostic predictor. Moreover, this further suggests the hypothesis that the measurement of NfL levels would be more likely to have prognostic value in patients with predominant LMN-related symptoms.

Our previous study proposed that assessing compound muscle action potential (CMAPs) amplitude is currently the most effective method to evaluate the impairments of LMNs and could be a prognostic indicator of disease progression in the early stages of ALS (8). Longitudinal studies have shown that blood NfL levels increase in the early symptom stage (lasting nearly 2 years) and then remain relatively stable over time (3, 9, 10). This study was performed to evaluate whether the serum levels of NfL were correlated with LMN axonal damage, as indicated by decreases in CMAP in the early stage of ALS.

Clarifying this issue is important, as previous studies have suggested that higher NfL levels were associated with poorer survival and more likely associated with a greater UMN disease burden but not LMN damage (9, 11, 12), offering the explanation that NfL may reflect corticospinal tract breakdown. These results were somewhat elusive when considered in conjunction, as studies widely suggested that LMN degeneration mainly dictates the prognosis, with a main contribution to non-invasive ventilation or death, whereas UMN involvement was poorly associated with survival (4, 13, 14). We hypothesize that NfL levels can be assumed to have prognostic value, mainly as a marker of LMN damage but not UMN involvement, and could effectively contribute to monitoring treatment efficacy.

## Materials and Methods

### Participants and Clinical Characterization

**Figure 1:**
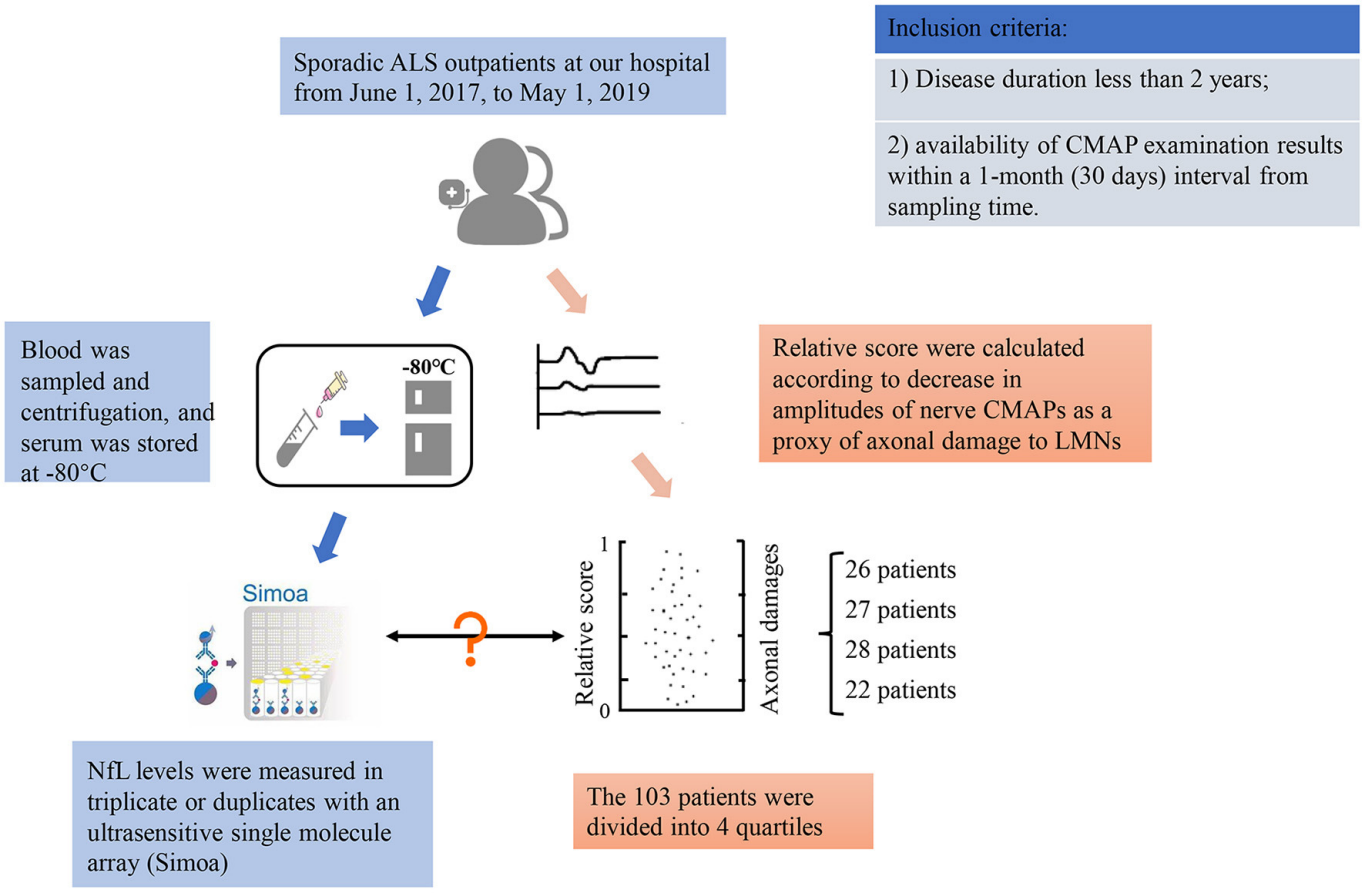
Study flow diagram.

This was a prospective, cross-sectional study of 103 sporadic ALS outpatients at Peking University Third Hospital, Beijing, China, from June 1, 2017, through May 1, 2019. For inclusion in the program, patients had to fulfill the following criteria: (1) sporadic ALS patients whose disease duration was less than 2 years from ALS onset to serum sampling and (2) who had CMAP examination results within a 1-month (30 days) interval from the sampling time. The study flow diagram is described in Figure 1. The 103 ALS patients were further subdivided by clinical subtype into 74 with typical ALS [clinically definite, probable, possible or lab-supported possible ALS according to the revised EI Escorial diagnostic criteria (15)], 15 with flail arm or leg syndrome (FAS or FLS), 1 with progressive muscular atrophy (PMA), and 13 with UMND ALS. The study was approved by the ethics committee of Peking University Third Hospital (PUTH) (IRB 00006761). Written informed consent was obtained from each patient, and all data were deidentified.

Each patient underwent a follow-up evaluation by telephone every 3 months. For all patients, baseline demographic information and clinical data were collected directly during the patient's first visit to PUTH and during follow-up evaluations. The demographic and clinical characteristics included age, sex, site of onset, age at onset, disease duration, phenotype of motor neuron disease, overall survival, and disease extent as assessed by the ALS Functional Rating Score–Revised (ALSFRS-R), which was performed closest to the time of blood sampling. The progression rate (ΔFS) until serum analysis was calculated as follows: ΔFS = (48–ALSFRS-R Score at Time of Diagnosis)/Duration From Onset to Diagnosis (Months) (16). The interval time between blood sampling and ALSFRS-R scoring was within 1 month (median: 0.1 months; range: 0–1 months). According to the quartile of ΔFS, slow (below the 25th percentile, <0.225), intermediate (between or equal to 0.225 and 0.800), and fast progressors (above the 75th percentile, >0.800) groups were defined.

### Sample Collection and Analysis

The 103 included patients underwent venipuncture at the Department of Neurology. Serum was obtained from the peripheral blood by centrifugation (2,664 g, 10 min, 12,000 g, 5 min) and stored within 2 h at −80°C until use; the samples were analyzed without any previous thaw-freeze cycle. Serum NfL levels were measured in triplicate or duplicate with an ultrasensitive single-molecule array (Simoa) platform provided by Quanterix (Lexington, MA, USA) as established previously (17–19). Measurements were performed on the fully automated HD-1 Analyzer (Quanterix) (20) instrument using the NF-L Beta kit from Quanterix, which employs an anti-NFL monoclonal antibody produced by UmanDiagnostics (Umeå, Sweden). The interassay coefficients of variation (CVs) were <10%. The linearity of the NFL assay was established (0–50,000 pg/mL). The researchers who performed the measurements were blinded to the clinical information (e.g., diagnostic category of any sample).

### CMAPs and Decreases

All ALS patients' electrophysiology examinations were performed within 1 month of blood sampling. The negative amplitudes of the CMAPs for motor nerve conduction in the median and ulnar nerves in the upper extremities as well as the peroneal and tibial nerves in the lower extremities of ALS patients were recorded (median nerve-waist, ulnar-waist, peroneal-ankle, tibial-ankle). These nerves were scored by the decrease in the amplitudes of each nerve CMAP- 0 (CMAP> =[X-2s]), 1 (50% [X-2s] < CMAP < [X-2s]), 2 (30% [X-2s] < CMAP <50% [X-2s]), 3 (CMAP < =30% [X-2s]), respectively. The values of X and S for different nerves and different patient age groups, which were applied in the electrophysiology examination room of Peking University Third Hospital, are listed in Supplementary Table S1. A decrease in the amplitude of the CMAPs of 8 nerves was summed (0–24) to reflect the overall LMN axonal damage of each ALS patient. For example, in one ALS patient, the bilateral ulnar nerves were scored as 1, the left median nerve was scored as 0, the right median nerve was scored as 2, the bilateral tibial nerves were scored as 3, the left peroneal nerve was scored as 3, and the right peroneal nerve was scored as 1. The absolute score of 8 nerves summed to 14.

However, only 4, 5, or 6 nerves underwent motor neuron conduction in approximately one-fourth of the ALS patients; thus, we used a relative score (relative score = absolute score/3 ^*^ number of nerves with data for motor neuron conduction; range: 0–1) as a proxy for the overall axonal damage of the LMNs. In the above example, the relative score was 1^*^2 + 0+2 + 3^*^2 + 3+1 = 14, 14 / (3^*^8) = 0.58.

The 103 patients were divided into 4 groups according to the relative score quartiles, with 22 patients in the first quartile of the relative score, 28 in the second quartile, 27 in the third quartile, and 26 in the fourth quartile (Supplementary Table S2).

Disease duration was defined as the time from symptom onset to serum sampling (months), and the time intervals between serum sampling and CMAP examination (days, within 30 days) for each ALS patient were calculated.

### Statistical Analysis

The NfL levels in the serum had an extremely left-skewed, non-normal distribution (Shapiro-Wilk test for normality *P* < 0.001). After natural logarithm transfor-mation, the data appeared to be normally distributed (Shapiro-Wilk test for normality *P* = 0.19) (Supplementary Figure S1). Thus, the log-transformed NfL (log [NfL]) concentrations were used for the graphical representation of data and one-way analysis of variance (ANOVA) with Tukey's multiple comparison test (*post-hoc* analysis).

The relative score for each patient also had an extremely left-skewed, non-normal distribution (Shapiro–Wilk test for normality *P* < 0.001). The correlation between the NfL levels and the relative score was tested using a second order coefficient partial correlation by keeping the ΔFS and King's College staging system (KCSS) stages constant. Correlations between the NfL levels and variables (serum conservation time, disease duration, ΔFS) were assessed using the Spearman correlation coefficient.

When the relative scores were treated as categorical variables using quartiles, the levels of serum log [NfL] were compared among the four ALS groups using one-way ANOVA with Tukey's multiple comparison test (*post-hoc* analysis). Comparisons of log [NfL] levels among patients with different disease progression rates (slow, mediate, fast) and different KCSS stages were assessed using one-way ANOVA with Tukey's multiple comparison test. The log [NfL] levels were compared among patients with different subtypes of ALS using one-way ANOVA with Tukey's multiple comparison test (*post-hoc* analysis).

The statistical analyses were performed using GraphPad Prism 5.0 (GraphPad Software, La Jolla, CA, USA) and IBM SPSS (Version 24). Statistical significance was set at a 2-sided *P* < 0.05.

## Results

The study included 103 patients with sporadic ALS (65 men [63.1%] and 38 women [36.9%]; median age, 55 years). The characteristics of the 103 ALS patients are summarized in Table 1. The sex and age distributions, serum conservation time, disease duration, BMI at baseline, and history of toxic exposure had no significant influence on the levels of serum NfL.

**Table 1:** Study participant characteristics: all participants.

	**N**	**Levels of NfL, pg/mL (Median, IQR)**	**Log [NfL] concentration (Mean, SD)**	***P*** **value**
All	103	55 (48–64)	1.77 (0.31)	NA
Sex				0.352
Male	65	62.3 (54.9)	1.75 (0.33)	
Female	38	68.6 (51.4)	1.81 (0.27)	
Age at sampling				0.548
<60 years	63	63.8 (44.8)	1.76 (0.31)	
≥60 years	40	62.5 (74.9)	1.80 (0.30)	
Disease duration				0.780
<12 months	55	70.3 (61.9)	1.77 (0.31)	
≥12 months	48	60.2 (47.6)	1.78 (0.30)	
ALS subtype				0.021
Typical ALS	74	65.4 (50.2)	1.80 (0.28)	
FAS or FLS	15	31.7 (54.7)	1.57 (0.39)	
PMA	1	138.6 (0)	2.14 (0)	
UMND	13	70.8 (57.6)	1.81 (0.27)	
Multiple LMN damage				<0.001
First quartile	22	49.1 (30.1–70.8)	1.62 (0.32)	
Second quartile	28	47.1 (33.2–81.9)	1.69 (0.29)	
Third quartile	27	73.3 (44.0–95.0)	1.82 (0.26)	
Fourth quartile	26	85.6 (70.3–149.5)	1.95 (0.28)	
KCSS stages				<0.0001
KCSS 1	53	49.05 (44.5)	1.64 (0.30)	
KCSS 2	30	85.6 (69.5)	1.92 (0.25)	
KCSS 3	12	66.8 (58.4)	1.85 (0.21)	
KCSS 4	4	146.5 (197.2)	2.16 (0.25)	
ΔFS				<0.001
Slow disease progression rate	24	37.6 (52.7)	1.6 (0.3)	
Intermediate progression rate	49	62.3 (52.6)	1.7 (0.3)	
Fast progression rate	23	103.6 (94.8)	2.0 (0.3)	
BMI at baseline*				0.154
<18.5	8	56.7 (32.8–85.7)	1.73 (0.2)	
18.5–23.9	46	71.9 (42.9–98.9)	1.82 (0.3)	
23.9–30	43	54.9 (28.3–87.2)	1.70 (0.32)	
≥30	5	70.3 (30.8–122.3)	1.79 (0.32)	
History of toxic exposure				0.799
Yes	16	67.8 (35.4–120.5)	1.77 (0.3)	
No	87	63.8 (39.2–92.4)	1.77 (0.4)	

*NfL, Neurofilament light chain; ALS, Amyotrophic lateral sclerosis; FAS, Flail arm syndrome; FLS, Flail leg syndrome; PMA, Progressive muscular atrophy; PLS, Primary lateral sclerosis; UMND, Upper motor neuron-dominant; KCSS, ΔFS = (48–ALSFRS-R score at time of diagnosis)/duration from onset to diagnosis (months). “toxic exposure” referred to recent histories of exposures to pollution, poisoning and pesticides*.

**Figure 2:**
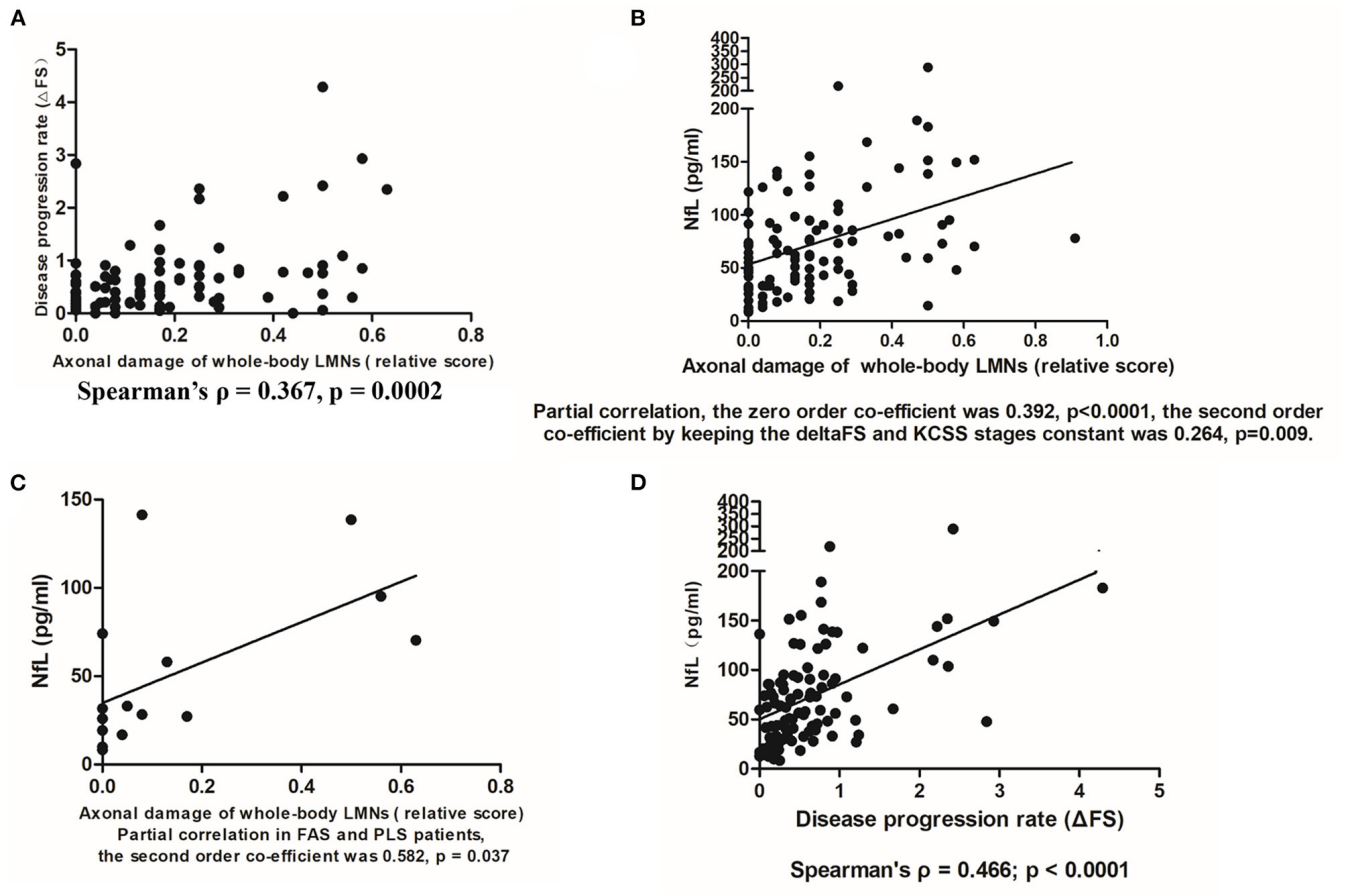
**(A)** Correlation between the relative score and disease progression rate (ΔFS) (*N* = 103). **(B)** The positive correlation between serum NfL levels and axonal damage of whole-body LMNs (indicated by the relative score) (*N* = 103). **(C)** The positive correlation between serum NfL levels and axonal damage to whole-body LMNs in FAS or FLS patients (*N* = 15). **(D)** The positive correlation between serum NfL levels and disease progression rates from disease onset to serum sampling (ΔFS) (*N* = 96).

The relative score was evaluated as a reliable indicator of axonal damage of LMNs; it was well correlated with the widely used disease progression proxy ΔFS (Spearman's ρ = 0.367, *p* = 0.0002) (Figure 2A). The NfL levels showed a significant positive correlation with the relative score (Figure 2B). Using partial correlation, the second order coefficient keeping the ΔFS and KCSS stages constant was 0.264 (*p* = 0.009). This analysis was re-performed again with 79 patients (79/103 = 76.7%) who had all 8 nerve CMAP recordings, and this re-analysis led to similar results (coefficient was 0.3, *p* = 0.008). Furthermore, when this correlation was estimated only among patients with disease subtypes that only involved LMNs—that is, FAS or FLS (15 patients)—the correlation coefficient largely increased (the second order coefficient was 0.582, *p* = 0.037), suggesting that the correlation between NfL levels and relative score was stronger (Figure 2C).

A significant correlation was found between ΔFS and the NfL level in ALS patients (Spearman's ρ = 0.466, *p* < 0.0001) (Figure 2D).

**Figure 3:**
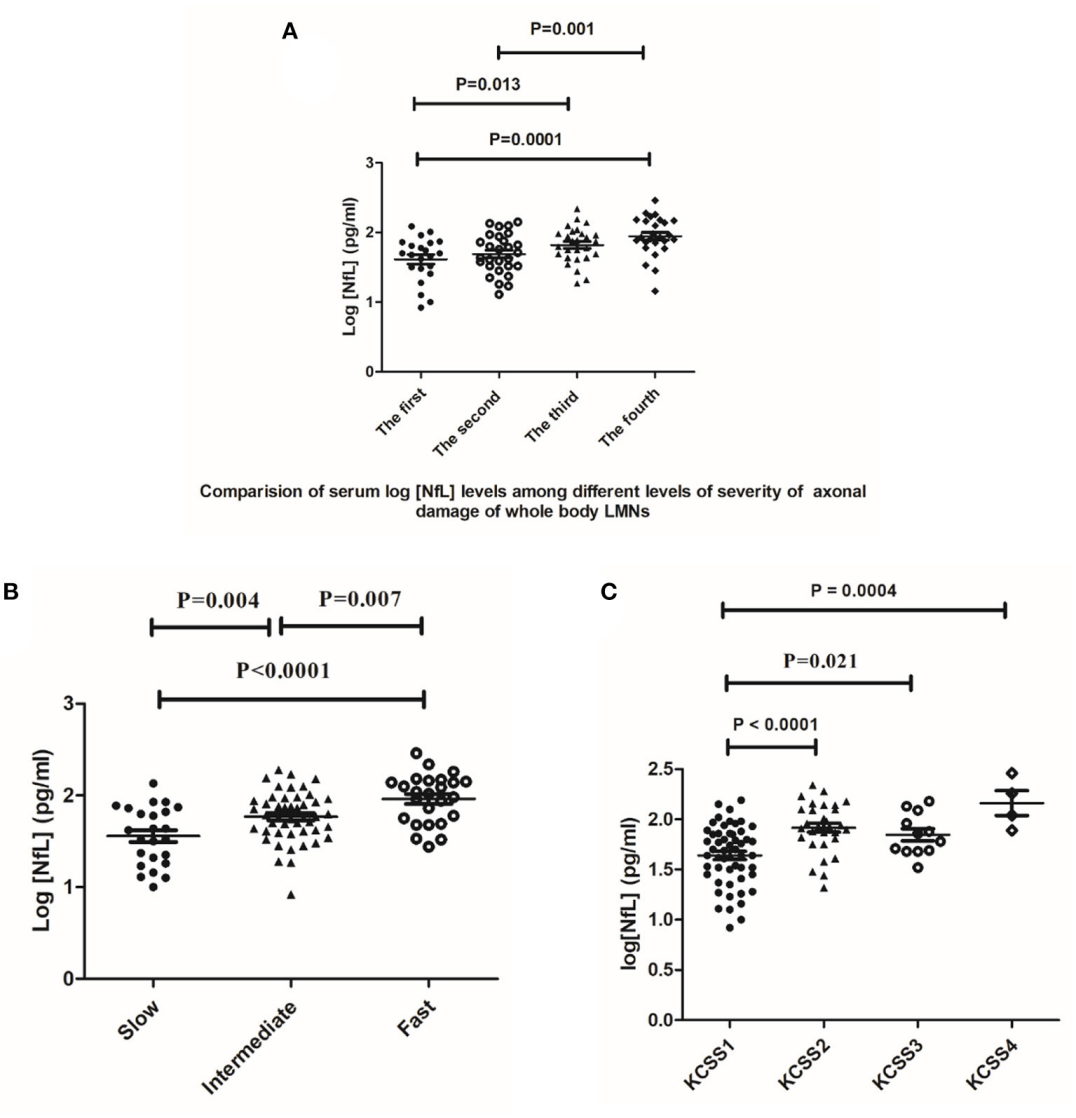
**(A)** Comparison of serum log[NfL] levels among four relative score quartiles of ALS. The mean value and scatter and dispersion of the observations are shown. Error bars indicate SD. *P* values were calculated using one-way ANOVA (*N* = 103). **(B)** Comparison of serum log[NfL] levels among the slow progression group, intermediate progression group, and fast progression group (*N* = 96). **(C)**. The serum levels of log[NfL] were positively correlated with the number of regions assessed by KCSS stages (*N* = 99). One-way ANOVA with *post-hoc* analysis by Tukey's multiple comparison test.

The levels of NfL differed among the four different quartile groups after natural logarithm transformation (F = 6.694, *P* = 0.0001) (Figure 3A). The serum log[NfL] concentrations were significantly higher in the fourth quartile of patients (mean [SD], 1.95 [0.28] pg/ml) than in the first quartile (mean [SD], 1.62 [0.32] pg/ml) and second quartile (mean [SD], 1.69 [0.29]; one-way ANOVA *p* < 0.0001 and *p* = 0.001, respectively). These differences remained significant when Tukey's multiple comparison test was used. In addition, patients in the third quartile (mean [SD], 1.82 [0.26]) of the relative score had higher serum log [NfL] concentrations than did patients in the first quartile, while this difference disappeared in *post-hoc* analysis (mean [SD], 1.62 [0.32] pg/ml; *P* = 0.013). The other clinical characteristics among the four groups are listed in Supplementary Table S2. The sex and age distributions, serum conservation time, disease duration, time interval between serum sampling and CMAP examination, ALS subtype distribution, and KCSS stages were similar among the four quartiles of patients.

Increasing disease progression rates (slow, intermediate, and fast groups) were associated with a stepwise increase in serum log[NfL] levels (mean 1.6 pg/ml, 1.7 pg/ml, and 2.0 pg/ml). Additionally, the three comparisons among the three groups were all significant (fast vs. slow, *P* < 0.0001; fast vs. intermediate, *P* = 0.007; intermediate vs. slow, *P* = 0.004) (Figure 3B and Table 1). All these differences remained significant when Tukey's multiple comparison test was used.

The serum levels of log[NfL] were positively correlated with the number of regions assessed by KCSS stages (KCSS 2 vs. KCSS 1; 1.92 vs. 1.64, *p* < 0.0001) (KCSS 4 vs. KCSS 1; 2.16 vs. 1.64, *p* = 0.0004) (KCSS 3 vs. KCSS 1; 1.85 vs. 1.64, *p* = 0.021) (Figure 3C). The differences (KCSS 2 vs. KCSS 1 and KCSS 4 vs. KCSS 1) remained significant when Tukey's multiple comparison test was used, while the significant difference between KCSS 3 vs. KCSS 1 disappeared.

**Figure 4:**
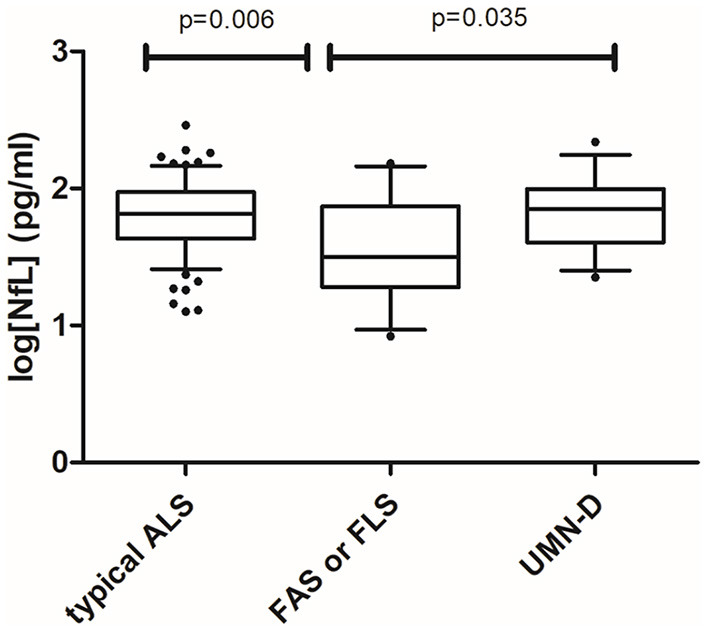
Pairwise comparisons of the typical ALS (*N* = 74), flail arm syndrome or flail leg syndrome (*N* = 15), and UMND groups (*N* = 13) were statistically significant, whereas there was no difference in log[NfL] levels between the typical ALS and UMND ALS groups. One-way ANOVA with *post-hoc* analysis by Tukey's multiple comparison test.

Pairwise comparisons among different subtypes showed that patients with typical ALS and those with UMN-D ALS had higher levels of NfL than did those with FAS or FLS(1.80 vs. 1.57, *p* = 0.006; 1.81 vs. 1.57, *p* = 0.035) (Figure 4). The difference between typical ALS and FAS or FLS remained significant, while the latter difference was non-significant in *post-hoc* analysis.

Log [NFL] levels were not significantly correlated with the serum conservation time (ρ = −0.03, *P* = 0.764) or the time from onset to serum sampling in patients with ALS (ρ = −0.188, *P* = 0.058) (Supplementary Figures S2A,B).

## Discussion

The serum NfL levels of patients with ALS were highly correlated with the extent of axonal degeneration of limb LMNs as assessed by the decrease in CMAPs. The study added direct and solid evidence to blood NfL concentrations that could mirror the extent of axonal damage of limb LMNs among ALS patients (12, 21). In future research, NfL levels in patients with predominant or even pure LMN signs will have progression and prognostic value. The use of NfL levels can have a profound effect on the selection of patients and monitoring of treatment efficacy in disease-modifying clinical trials.

This study showed that serum NfL levels correlated with the disease progression rate as assessed by the decline in the ALSFRS-R and KCSS stages. These results were consistent with those of previous studies showing that serum or CSF NfL levels correlated with the parameters of disease severity, such as the decline on the ALSFRS-R (3, 9, 12, 22). Our results are also in line with the correlation between serum NfL levels and the number of regions displaying UMN and/or LMN degeneration (9). Thus, the fact that NfL serum or plasma levels seem likely to reflect the axonal loss of LMNs in ALS patients supports the idea that NfL levels will perform well with regard to predicting disease progression and survival.

We found that serum NfL concentrations in typical ALS patients are significantly higher than those in FAS or FLS patients. The actual concentration of serum NfL mainly reflected the severity and speed of axonal damage, which indicats a state of dynamic equilibrium in which protein synthesis, protein release into blood, turnover, and immune reactions are balanced with degradation. Although recognized as pure LMN axonal damage, the slower disease progression of FAS or FLS may be a plausible explanation for lower serum NfL levels.

One could point out that a proportion of blood NfL levels may originate from the CSF, as CSF levels were seen to be well correlated with that those in serum, (2, 10, 11) suggesting that at least part of the NfL chain could breach the blood–brain barrier. However, the serum NfL level is a proposed biomarker of pure LMN damage in diseases such as inherited peripheral neuropathy (23), vasculitic neuropathy (24), and chronic inflammatory demyelinating polyradiculoneuropathy (CIDP) (25), suggesting that body fluid NfL levels permit estimation of the amount of axonal degeneration. Furthermore, patients with primary lateral sclerosis (PLS) and hereditary spastic paraplegia (HSP) had relatively low levels of serum NfL, which may be partly due to the slow rate of UMN degeneration in these disorders (9, 26). These results also indicated that blood NfL originating from the CNS was limited.

Another piece of evidence supporting the concept that the increase in the serum levels of NfL is due to protein release after axonal degeneration is that the expression of NfL maintains steady levels throughout the disease course (3). Notwithstanding its strengths, this study does have limitations. First, the cross-sectional studies play a limited role in explaining the relationship between serum NfL levels and axonal damage of LMNs. Second, a previous study showed that the levels of antibodies against NF proteins in the plasma were significantly higher in ALS individuals than in healthy controls (27). Thus, investigating the immune response to NfL protein release, aggregation and abnormal phosphorylation may be a better strategy for biomarker discovery in ALS. Third, only 4, 5, or 6 nerves were subjected to motor neuron conduction in approximately one-fourth of the ALS patients, and this was usually conducted in other hospitals. We did not know the exact reason why some nerves were not subjected to CMAP measurement, but we reasonably hypothesized that the “exempt” nerves were usually omitted because the corresponding limbs were free from the disease symptoms. The healthy “exempt” nerves probably had very little influence on the level of serum NfL, which was confirmed by the new results and was similar to previous results. Finally, evaluation of NfL concentrations in CSF and blood from the same patient would be important to validate our hypothesis and might provide additional information about the use of NfL as a biomarker of ALS. Further study designs should take this into consideration.

## Data Availability Statement

The raw data supporting the conclusions of this article will be made available by the authors, without undue reservation.

## Ethics Statement

The studies involving human participants were reviewed and approved by the Ethics Committee of Peking University Third Hospital (PUTH) (IRB 00006761). The patients/participants provided their written informed consent to participate in this study.

## Author Contributions

LZ had full access to all the data in the study, takes responsibility for the integrity of the data, the accuracy of the data analysis, drafting of the manuscript, and statistical analysis. DF: concept and design. LZ, TJ, CW, and XL: acquisition, analysis, or interpretation of data. All authors: critical revision of the manuscript for important intellectual content. SZ, NZ, and LT: administrative, technical, or material support.

## Funding

This study was supported by the National Natural Science Foundation of China (81030019 and 81873784) and Clinical Cohort Construction Program of Peking University Third Hospital, Grant/Award Number: BYSYDL2019002.

## Conflict of Interest

The authors declare that the research was conducted in the absence of any commercial or financial relationships that could be construed as a potential conflict of interest.

## Publisher's Note

All claims expressed in this article are solely those of the authors and do not necessarily represent those of their affiliated organizations, or those of the publisher, the editors and the reviewers. Any product that may be evaluated in this article, or claim that may be made by its manufacturer, is not guaranteed or endorsed by the publisher.
