# Supplementary material for: Serum Neurofilament Light Chain Levels May Be a Marker of Lower Motor Neuron Damage in Amyotrophic Lateral Sclerosis
Source: Front Neurol. 2022 Feb 23;13:833507. doi: 10.3389/fneur.2022.833507 (PMC8905596; doi:10.3389/fneur.2022.833507)
Supplement: Supplementary file 1 [file Data_Sheet_1.docx]

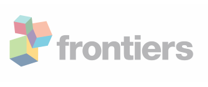


**Supplementary Material**


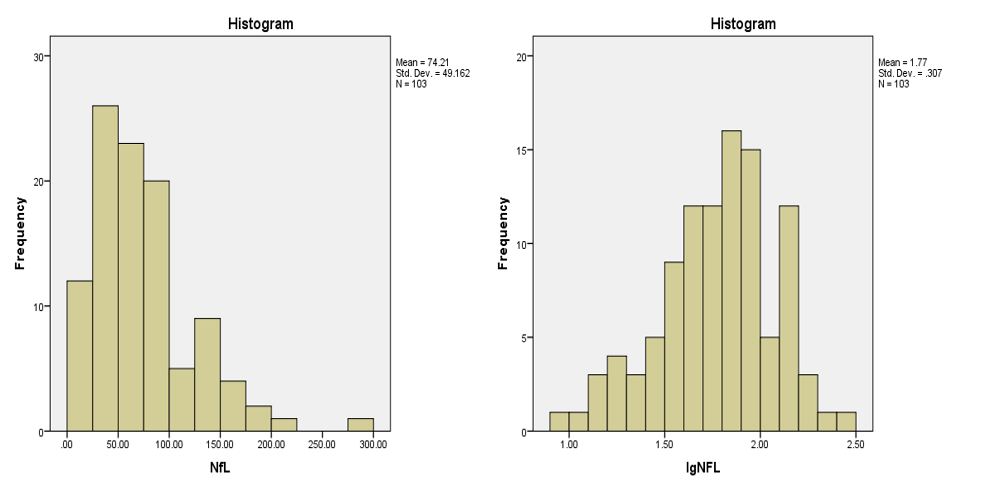


**Figure S1.** Natural logarithm transformation of serum NfL concentration with subsequent normal distribution of data.


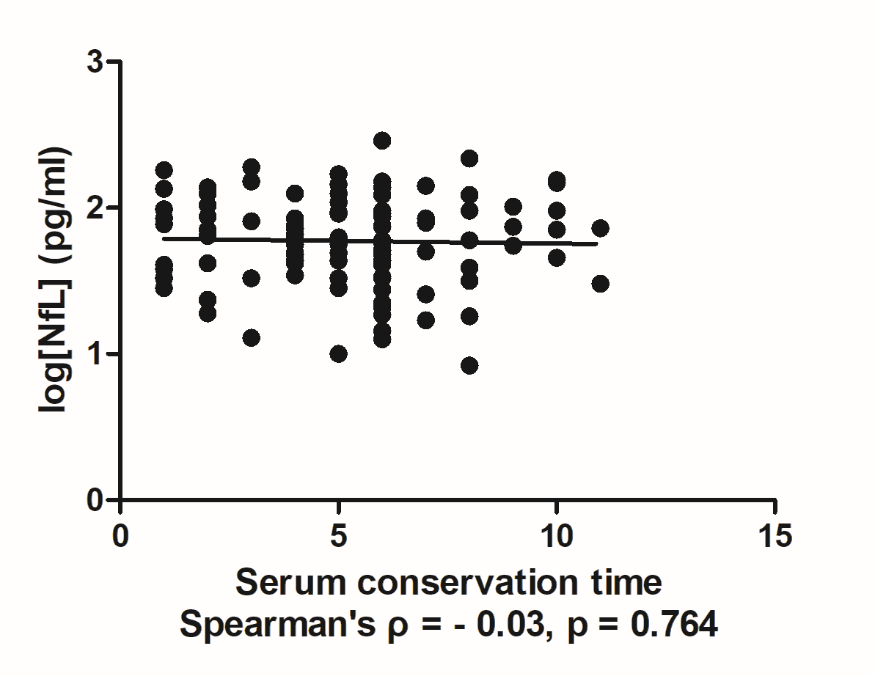

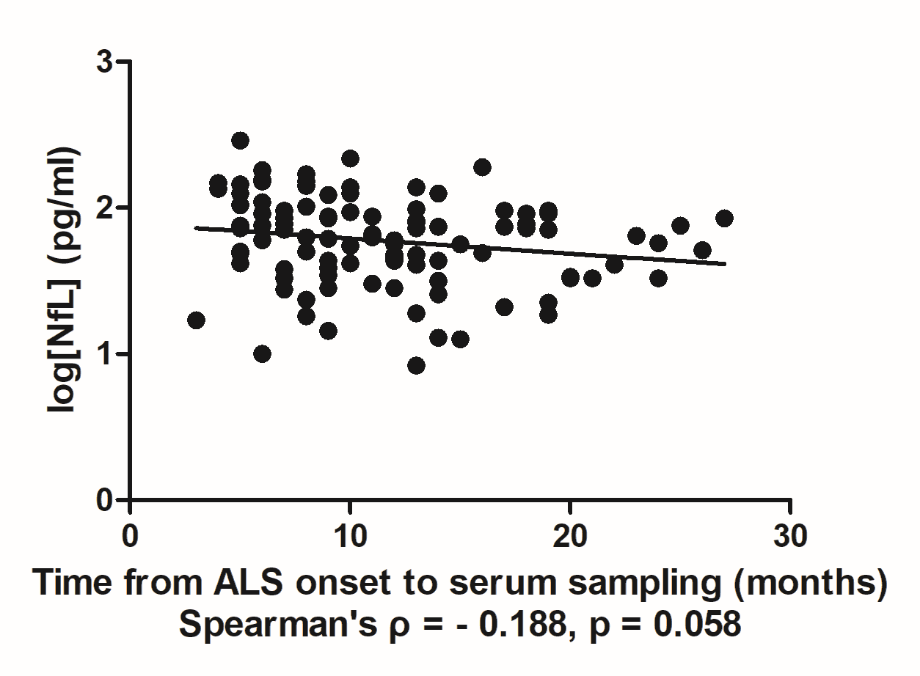


**B**

**A**

**Figure S2A.** Effect of serum conservation time on the concentration of NfL in ALS patients. **B.** Effect of time from ALS onset to serum sampling on the concentration in ALS patients.

**Table S1.** The values of X and S for different nerves and different patient ages measured in the electrophysiology examination room of Peking University Third Hospital.

|  | Age group | χ±s （mV） | 0 (CMAP> [X-2s]) | 1 (50% [X-2s] < CMAP ≤[X-2s]) | 2 (30% [X-2s] < CMAP ≤ 50% [X-2s]) | 3 (CMAP ≤ 30% [X-2s]) |
| --- | --- | --- | --- | --- | --- | --- |
| Median nerves | ＜35-45 | 11.63±3.25 | 5.13 | ≤5.13 | ≤2.57 | ≤1.59 |
|  | 45-64 | 10.38±3.04 | 4.3 | ≤4.3 | ≤2.15 | ≤1.29 |
|  | 65-74 | 9.08±2.54 | 4 | ≤4 | ≤2 | ≤1.2 |
| Ulnar nerves | ＜35-45 | 11.71±3.59 | 4.53 | ≤4.53 | ≤2.265 | ≤1.359 |
|  | 45-64 | 9.11±2.49 | 4.13 | ≤4.13 | ≤2.065 | ≤ 1. 239 |
|  | 65-74 | 10.28±3.28 | 3.72 | ≤3.72 | ≤1.86 | ≤ 1.116 |
| Tibial nerves | 11-78 | 5.8±0.95 | 3.9 | ≤3.9 | ≤1.95 | ≤1.17 |
| Peroneal nerves | 16-86 | 5.1±1.15 | 2.8 | ≤2.8 | ≤ 1.4 | ≤0.84 |

Note: CMAP: compound muscle action potentials. The values of X and s were obtained from healthy individuals in different age groups at 25 °C ambient temperature.

**Table S2.** Characteristics of the clinical parameters among the four groups.

|  | **First quartile** | **Second quartile** | **Third quartile** | **Fourth quartile** | **All** | **P value** |
| --- | --- | --- | --- | --- | --- | --- |
| No. Patients | 22 | 28 | 27 | 26 | 103 | NA |
| Male/female | 15/7 | 21/7 | 14/13 | 15/11 | 65/38 | 0.291 |
| Levels of NfL, pg/mL (median, IQR) | 49.1 (30.1-70.8) | 47.1 (33.2-81.9) | 73.3 (44.0-95.0) | 85.6 (70.3-149.5) | 63.8 (39.3-94.6) | 0.001 |
| Log [NfL] concentration (mean, SD) | 1.62 （0.32） | 1.69 （0.29） | 1.82 （0.26） | 1.95 （0.28） | 1.77 （0.31） | <0.001 |
| Median (IQR) |  |  |  |  |  |  |
| Age at blood sampling | 56 （47-60） | 56 （47.5-63） | 55 （49-66） | 55 （47-64） | 55 （48-64） | 0.815 |
| Serum conservation time, mo | 6 （4-8） | 5 （2-6） | 5 （4-6） | 6 （4-6） | 5 （4-6） | 0.152 |
| Time interval, days | 10 （8-16） | 9.5 （5-12.5） | 7 （5-12） | 8 （6-12） | 9 （5-13） | 0.63 |
| ALS subtype |  |  |  |  |  | 0.137 |
| Typical ALS | 11 | 20 | 22 | 21 | 74 |  |
| FAS or FLS | 6 | 5 | 1 | 3 | 15 |  |
| PMA | 0 | 0 | 0 | 1 | 1 |  |
| PLS | 0 | 0 | 0 | 0 | 0 |  |
| UMND | 5 | 3 | 4 | 1 | 13 |  |
| Disease duration (median, IQR) | 12 （9-18） | 11.5 （7-14.5） | 9 （7-14） | 10 （8-14） | 11 （7-15） | 0.823 |
| deltaFS | 0.34 (0.17-0.58) | 0.37 (0.19-0.63) | 0.52 (0.33-0.95) | 0.77 (0.3-1.48) | 0.49 (0.23-0.80) | 0.008 |
| KCSS stages* (n) |  |  |  |  |  | 0.128 |
| 1 | 13 | 19 | 13 | 8 | 53 |  |
| 2 | 6 | 4 | 10 | 10 | 30 |  |
| 3 | 2 | 3 | 3 | 4 | 12 |  |
| 4 | 0 | 0 | 1 | 3 | 4 |  |

Note：* Four patients did not have available KCSS stage data.
